# A Tudor Domain Protein SPINDLIN1 Interacts with the mRNA-Binding Protein SERBP1 and Is Involved in Mouse Oocyte Meiotic Resumption

**DOI:** 10.1371/journal.pone.0069764

**Published:** 2013-07-22

**Authors:** Ting Gang Chew, Anne Peaston, Ai Khim Lim, Chanchao Lorthongpanich, Barbara B. Knowles, Davor Solter

**Affiliations:** 1 Mammalian Development Laboratory, Institute of Medical Biology, A-STAR, Singapore, Singapore; 2 University of Adelaide, School of Animal and Veterinary Sciences, Roseworthy, Australia; 3 Duke-NUS Graduate Medical School, Singapore, Singapore; 4 Department of Biochemistry, Yong Loo Lin School of Medicine, National University of Singapore, Singapore, Singapore; Institute of Zoology, Chinese Academy of Sciences, China

## Abstract

Mammalian oocytes are arrested at prophase I of meiosis, and resume meiosis prior to ovulation. Coordination of meiotic arrest and resumption is partly dependent on the post-transcriptional regulation of maternal transcripts. Here, we report that, SPINDLIN1 (SPIN1), a maternal protein containing Tudor-like domains, interacts with a known mRNA-binding protein SERBP1, and is involved in regulating maternal transcripts to control meiotic resumption. Mouse oocytes deficient for *Spin1* undergo normal folliculogenesis, but are defective in resuming meiosis. SPIN1, via its Tudor-like domain, forms a ribonucleoprotein complex with SERBP1, and regulating mRNA stability and/or translation. The mRNA for the cAMP-degrading enzyme, PDE3A, is reduced in *Spin1* mutant oocytes, possibly contributing to meiotic arrest. Our study demonstrates that *Spin1* regulates maternal transcripts post-transcriptionally and is involved in meiotic resumption.

## Introduction

During fetal development in mammals, the female germ cell enters meiosis and arrests at meiotic prophase I with a distinctive germinal vesicle (GV) in the cell center. After a long period of meiotic arrest and oocyte growth, the fully grown oocyte resumes meiosis upon the stimulation of hormones during puberty [1]. The oocyte undergoes germinal vesicle breakdown (GVBD) and reduces its chromosome to the haploid number, which is retained in the egg pronucleus. The oocyte then arrests at the metaphase of second meiotic division, and awaits fertilization to complete meiosis [2].

The oocyte ceases transcription when it is fully grown [3]. The resumption and completion of meiosis are highly dependent on maternal mRNAs and proteins stored during oocyte growth [4], [5], [6], [7]. Maternal mRNAs form ribonucleoprotein (RNP) complexes with RNA-binding proteins, which stabilize the maternal mRNAs and the mRNAs are translated into proteins in a just-in-time fashion [8], [9]. The mRNAs of several cell cycle regulatory proteins are stabilized and timely translated during the transition of meiotic arrest to meiotic resumption [6], [7], [10].

Most RNA-binding proteins are post-translationally modified by protein arginine methylation during the formation of RNP complexes [11]. The methylated RNA-binding protein is recognized and bound by proteins containing Tudor domains [12]. In several organisms, proteins containing Tudor domains are required for proper formation and function of RNP complexes during germline development [13]. Interestingly, in the mouse oocyte, a protein containing multiple Tudor-like domains, SPINDLIN1 (SPIN1), has been identified as a highly expressed maternal protein and has been suggested to play a role in meiosis [14], [15]. Although a role in somatic cells has been demonstrated [16], [17], [18], how SPIN1, which is highly expressed in mouse oocytes, functions in the oocyte during meiosis remains largely unknown.

In this study, we show that meiotic resumption is defective in mouse oocytes deficient in SPIN1 and maternal transcript abundance is affected. SPIN1 appears to exert it function by interacting with the mRNA-binding protein SERBP1.

## Materials and Methods

### Animal Maintenance and Embryo Culture

Mice for this study were maintained in a specific-pathogen free facility provided by the Singapore Biological Resource Centre (Biopolis), a member of Singapore A*STAR's (Agency for Science, Technology and Research) Biomedical Sciences Institutes with the approval of the Singapore A*STAR Genetic Modification Advisory Committee. The experiments were approved by the Singapore A*STAR Institutional Animal Care and Use Committee (IACUC), under the Singapore A*STAR IACUC number 110673. The *Spin1* genetrap embryonic stem cell clone RRZ449 obtained from BayGenomics was used to generate the genetrap mouse in the Jackson Laboratory transgenic mouse service. The genetrap mouse was subsequently backcrossed for ten generations to C57BL/6J (B6.Cg-Spin1GT^RRZ449Byg^). Fully grown oocytes were recovered from ovaries by puncturing follicles with a 30G needle, denuded, and cultured in Eagle’s minimal essential medium (Gibco) containing 0.23 mM sodium pyruvate and 3 mg/ml bovine serum albumin (BSA). To prevent spontaneous meiotic resumption, 0.2 mM 3-isobutyl-1-methylxanthine (IBMX) was added to the medium. To obtain pre-implantation embryos at different stages, zygotes were harvested from oviducts of superovulated (C57BL/6× DBA2; B6D2) F1 female mice mated with B6D2 F1 male mice, and cultured in KSOM medium (Chemicon) to the desired embryonic stage.

### Tissue Transplantation

The fetal gonad was dissected from E18.5 fetuses of time-mated *Spin1* genetrap heterozygotes and kept at room temperature in PBS with 10% fetal bovine serum (FBS) until transplanted under the kidney capsule of C57BL/6 mice as described [19]. After 20 days, mice bearing the transplanted gonad in the kidney capsule were intraperitoneally injected with 5 I.U. of equine chorionic gonadotropin (eCG, also known as pregnant mare serum gonadotropin) and the gonad was dissected 44 hours post-injection.

### Mammalian Cell Culture and Transfection

HEK293T cells were cultured in DMEM (Gibco) supplemented with 10% FBS. Plasmids prepared using QIAGEN maxiprep kit were transfected into HEK293T cells with Lipofectamine 2000 (Life Technologies).

### Molecular Cloning

To fuse mouse *Spin1*, *Serbp1*, and *Habp4* with Myc or HA tags, the cDNAs were amplified and cloned into pCMV6-AN (Origene). Primers used are listed in Table S1. Site-directed mutagenesis of *Spin1* was performed using a PCR-based method with overlapping primers, Vent DNA Polymerase (NEB) and DpnI restriction enzyme (NEB). Table S2 lists the primer sequences used in site-directed mutagenesis. To generate DNA constructs for the luciferase reporter assay, a DNA fragment containing 134 nucleotides of the 3′ UTR of rat *Serpine1* was cloned into pmirGLO vector (Promega) downstream of a luciferase. Primers used in pmirGLO cloning are listed in Table S3.

### Reverse Transcription-PCR and Real-time Quantitative PCR

Total RNAs of fully grown oocytes were purified using a PicoPure RNA Isolation Kit (Arcturus Bioscience), and reverse transcribed to cDNAs using the SuperScript III reverse transcriptase (Life Technologies) and oligo-dT. The synthesized cDNA was amplified in a sequence-specific manner, using the TaqMan PreAmp Master mix kit (Applied Biosystems), prior to analysis with the Universal Probe Library (Roche Diagnostic) for gene expression assays. All real time qPCR was run on the Prism 7900HT Sequence Detection System 2.2 (ABI), and Ct values were calculated by the system software, and normalized to the Ct value of *Gapdh*. Primers used in the real time qPCR are listed in Table S4.

### Immunoprecipitation Assay and Western Blotting

Cell extracts were solubilized in lysis buffer containing 25 mM Tris-Cl pH7.4, 150 mM NaCl, 1% Triton X-100, and complete protease inhibitors (Roche Diagnostics), and clarified by centrifugation at 14,000 rpm for 10 min at 4°C. To immunoprecipitate protein complexes, soluble proteins were incubated with antibodies pre-bound to Protein A Sepharose CL-4B beads (GE Healthcare) for 8 hours at 4°C. After several washes with buffer containing 1% Triton X-100, the beads were resuspended in SDS-PAGE loading buffer and heated at 95°C for 5 min. The Protein A-Sepharose beads were spun down, 14,000 rpm for 5 min, and the supernatants were subjected to SDS-PAGE. To detect SPIN1, SERBP1, MYC-tagged proteins, or HA-tagged proteins, antibodies recognizing SPIN1, SERBP1 (Santa Cruz), MYC (Santa Cruz), or HA (Sigma) were used to probe the PVDF membranes containing separated proteins. Western blotting was performed as described [20].

### Immunocytochemistry

Prior to fixation, zonae pellucidae were removed with acidic Tyrode’s solution (Chemicon). Oocytes were fixed in a prewarmed solution, containing 100 mM HEPES (pH7.0), 50 mM EGTA, 10 mM MgSO4, 0.2% Triton X-100, 3.7% formaldehyde (Sigma), for 30 minutes at room temperature. Fixed oocytes were then permeabilized for 2 hours in PBS with 0.2% Triton X-100, and then incubated in PBS with 10% FBS for at least 30 minutes. Interval washes were done in PBS with 0.1% polyvinylpyrrolidone. Incubation with primary antibody was overnight at 4°C, followed by one hour incubation with secondary antibody at room temperature. The rabbit SPIN1 antibody and the mouse tubulin antibody (Sigma) were used at 1∶100 dilutions as primary antibodies. Hoechst 33342 dye was used to stain DNA.

### Histology and Immunohistochemistry

For immunodetection, dissected ovaries were first fixed using PBS with 4% formaldehyde at 4°C for 12 hours, then processed into tissue blocks and cryo-sectioned. The tissue sections were incubated in PBS with 10% FBS for at least 30 minutes, and then with primary antibodies recognizing SPIN1 and SERBP1 for 4 hours at room temperature. Secondary antibodies conjugated with Alexa Fluor-488 or 594 were added after three washes with PBS containing 0.1% Tween 20. DNA was stained using Hoechst 33342. To reveal the histological structure of the gonad grafted under the kidney capsule, the tissue was processed into wax blocks, and then sectioned and stained with hematoxylin and eosin.

### Confocal Microscopy

Fixed oocytes were imaged on glass bottom dishes (WillCo Wells) with a Zeiss LSM510 confocal microscope equipped with a 40X EC Plan-NeoFluor 1.3 NA oil immersion objective lens. Images were acquired using LSM510 software and analyzed using ImageJ (NIH).

### Luciferase Reporter Assay

The luciferase reporter assay was done using the Dual-Luciferase Reporter Assay System (Promega). The pmirGLO plasmids, containing the 3′UTR of *Serpine1* or *Pde3A* were co-transfected into HEK293T cells with pCMV6-AN plasmids containing *Myc*, *Myc-Spin1*, *Myc-Spin1Y155F*, or *HA-Serbp1*, respectively. The luciferase activity of empty vectors pmirGLO and pCMV6-AN was used to normalize the expression level.

### Yeast Two-hybrid Screening

The CytoTrap Yeast Two-hybrid system (Agilent Technologies) was used to screen for interacting proteins of SPIN1. The *Spin1* cDNA was first cloned into an pSOS vector, and co-transformed with a mouse ovarian cDNA library. Yeast transformation was performed using Yeastmaker Yeast Transformation System 2 (Takara Bio).

### Bioinformatics and Statistical Analyses

The three-dimensional structure of human SPIN1 was visualized and edited using Molmol. The protein structure was downloaded from Protein Data Bank (PDB). Statistical significance was determined using Student’s t-test where appropriate. Calculations of average, standard error of the mean, and statistical significance, were done using Prism 5.03 (GraphPad).

## Results

### Ovarian Folliculogenesis and Oocyte Growth Appear Normal in *Spin1* Mutant Ovary

To understand the physiological roles of SPIN1, we characterized a mouse genetrap line in which a cassette containing a splice acceptor site was inserted in the intron between exon 3 and 4 of the *Spin1* genomic locus (Figure S1A). Heterozygous mice containing a *Spin1* allele inserted by the genetrap cassette were viable and fertile. When the *Spin1* genetrap heterozygotes were intercrossed, only wild type and heterozygous offsprings, but no homozygous mice, were obtained at weaning (Table 1). Further analysis showed that homozygous genetrap pups are present at E18.5 but exhibit early post-natal death, with homozygous pups dying within 2 days of birth (Figure S1B). Characterization of *Spin1* genetrap homozygous fetal gonads at E18.5 shows that *Spin1* mRNA and proteins are barely detectable in these tissues, indicating that the *Spin1* genetrap homozygote is a null allele for *Spin1* function (hereafter refer to as *Spin1* mutant) (Figure S1C and D).

**Table 1:** Summary of the genetic crosses of Spin1-GT heterozygous.

Ratio	+/+ (wild type)	Spin1-GT/+	Spin1-GT/Spin1-GT
**Expected**	1	2	1
**Experimental**	1 (28 pups)	1.786 (50 pups)	0 (0 pup)

Based on analyses of 19 litters from 6 mating pairs, ∼4 pups/litter.

**Figure 1:**
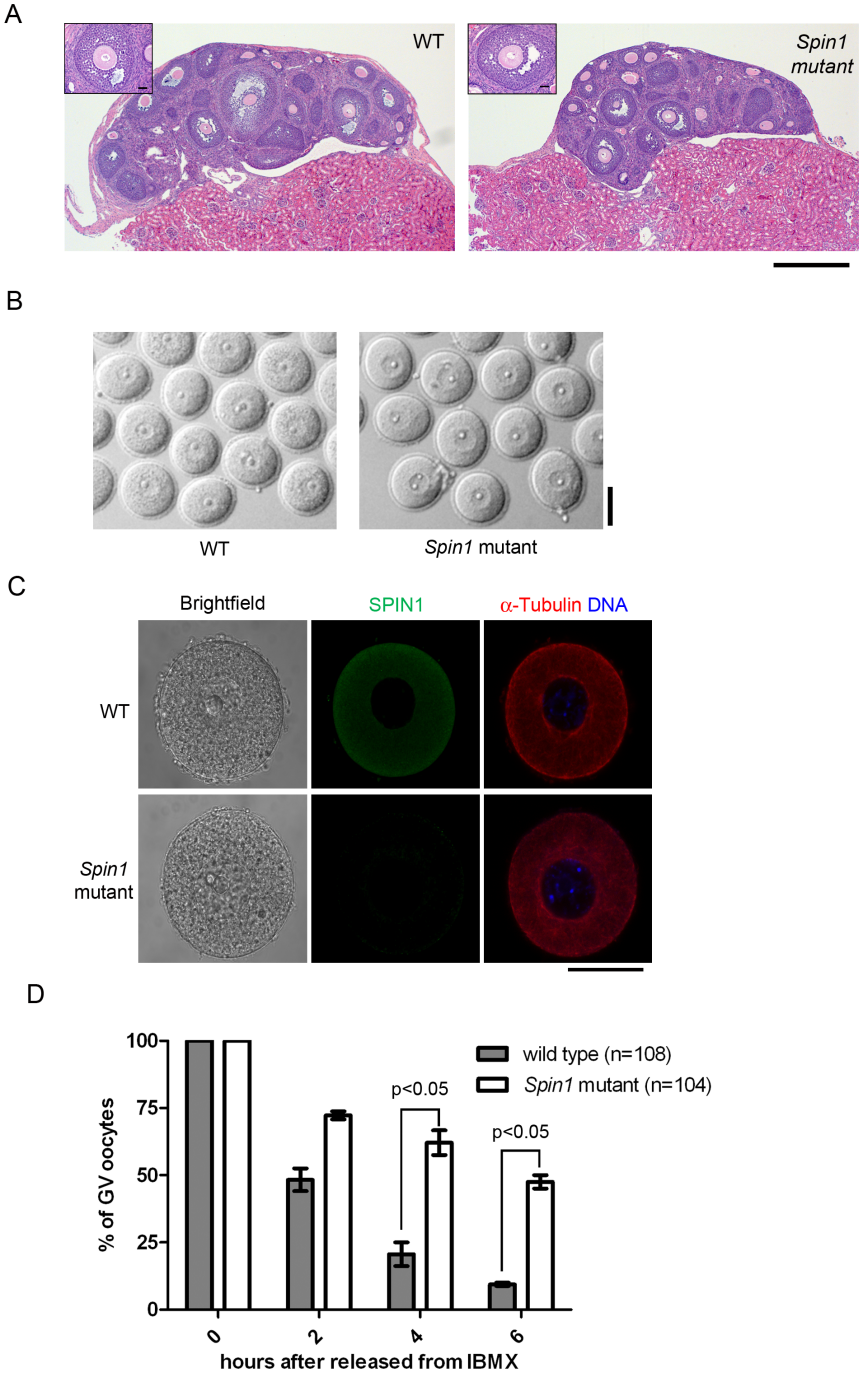
*Spin1* mutant undergoes normal folliculogenesis and oocyte growth, but exhibit defective meiotic resumption. (A) Haematoxylin and eosin staining of ovarian sections grafted under the kidney capsules. Left panel: wild type; right panel: *Spin1* mutant. Scale bar represents 500µm. (B) Fully grown oocytes harvested from wild type and *Spin1* mutant ovaries after stimulation by PMSG. Scale bar represents 50µm. (C) Immunofluorescence staining of fully grown oocytes by anti-SPIN1 (green) and anti-Tubulin (red) antibodies. DNA (blue) was visualized by Hoechst 33342 dye. Scale bar represents 50µm. (D) Quantification of the percentage of fully grown oocytes with GV after release from the phosphodiesterase inhibitor IBMX. Data are mean ± SEM, Student’s t-tests.

Since *Spin1* mutants exhibit early post-natal lethality, we grafted ovaries of E18.5 *Spin1* mutant and wild type littermates under the kidney capsules of C57BL6/J adult mice, to study SPIN1 function during oocyte growth and maturation. The procedure of grafting ovaries under adult kidney capsules has previously been shown to support ovarian growth and development [21]. Grafted ovaries were removed and sectioned after 21 days following stimulation by PMSG. Staining the ovarian sections showed that follicles at different stages including pre-antral follicles, early antral follicles, and antral follicles were present in the *Spin1* mutant ovary, similar to ovarian follicles of wild type controls (Figure 1A). We then punctured mutant and wild type ovaries to harvest fully grown oocytes (Figure 1B), and immunostained the oocytes with antibodies recognizing SPIN1 and β-tubulin. SPIN1 was only detected in wild type control oocytes, but not in *Spin1* mutant oocytes. The microtubules displayed interphase-like distribution and were indistinguishable between mutant and wild type control oocytes (Figure 1C).

Taken together, we show that *Spin1* genetrap homozygous mice are deficient in *Spin1* mRNA and proteins, but follicular development and oocyte growth are unaffected by the absence of SPIN1.

### *Spin1* Mutant FGOs are Defective in Resuming Meiosis

Fully grown oocytes arrested at prophase I, resume meiosis when released from ovarian follicles. To examine whether SPIN1 plays a role in meiotic resumption, we first harvested oocytes containing intact GV from *Spin1* mutant and wild type control ovarian follicles in medium consisting of IBMX, a phosphodiesterase inhibitor that prevents spontaneous GV breakdown. The denuded oocytes were then released into IBMX-free medium, and the ability of oocytes to resume meiosis was scored by GVBD. Upon release into IBMX-free medium, control oocytes underwent GV breakdown efficiently, in contrast to *Spin1* mutant oocytes. After 6 hours incubation in IBMX-free medium, 50% of *Spin1* mutant oocytes remained as GV oocytes, whereas less than 10% of control oocytes retained a GV (Figure 1D). Overnight incubation in IBMX-free medium showed that a significant number of *Spin1* mutant oocytes (around 50%) failed to resume meiosis as compared to control oocytes (less than 5%). Our findings suggest SPIN1 plays a role in meiotic resumption of mouse oocytes. The fact that half of the *Spin1* mutant oocytes retained the ability to resume meiosis suggested that *Spin1* functions in oocytes are partly complemented by other mechanisms involved in meiotic resumption [2].

### SPIN1 Interacts with Hyaluronan/mRNA-binding Protein Family Members: SERBP1 and HABP4

**Figure 2:**
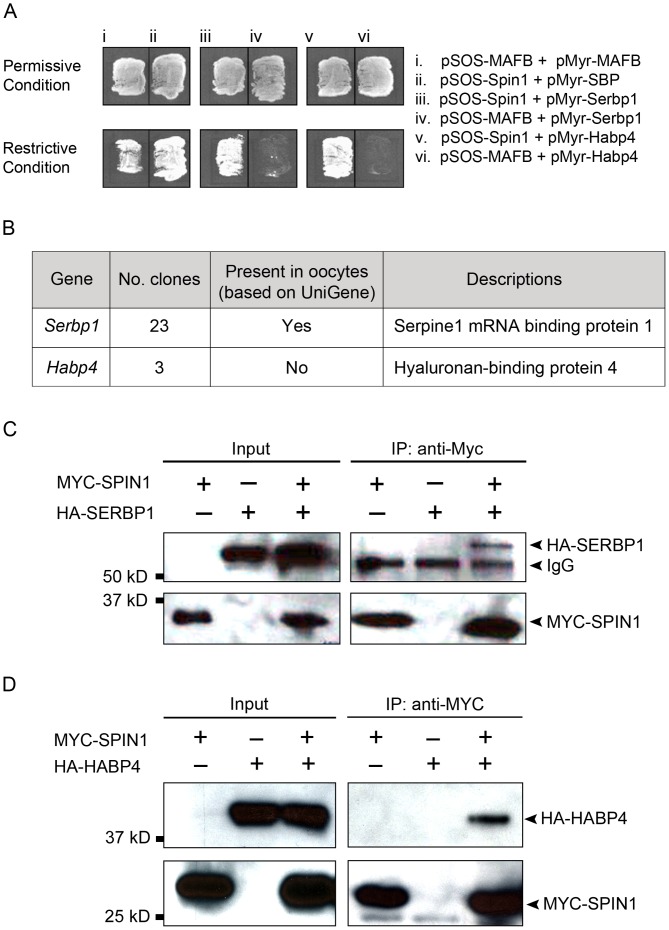
Yeast two-hybrid screening identifies hyaluronan/mRNA-binding protein family members: SERBP1 and HABP4 as binding proteins of SPIN1. (A) Yeast cells transformed with different plasmids were grown on selective medium under permissive condition (24°C) and restrictive condition (37°C). i and ii are positive controls, iv and vi are negative controls, iii and v are yeast cells with positive clones from the yeast two-hybrid screening, co-transformed with bait plasmid containing *Spin1*. (B) Summary of the yeast two-hybrid screening. (C) HA-tagged SERBP1 is co-immunoprecipitated with MYC-tagged SPIN1 using MYC-antibody and extract from HEK293T cells expressing HA-SERBP1 and MYC-SPIN1. (D) HA-tagged HABP4 is co-immunoprecipitated with MYC-SPIN1 using MYC-antibody and extract from HEK293T cells expressing HA-HABP4 and MYC-SPIN1.

To understand the molecular functions of SPIN1 in regulating meiotic resumption, we aimed to identify binding partners of SPIN1. We used the CytoTrap yeast two-hybrid system to screen the mouse ovarian cDNA library for proteins that interact with SPIN1. Out of 151 yeast colonies that grew on the selective medium at restrictive temperature after yeast transformation, 26 colonies displayed reproducible growth after two rounds of selection (Figure 2A and B). Sequencing analysis of the recovered cDNA clones revealed 23 clones encoded *Serpine1 RNA binding protein* (*Serbp1)*, and 3 clones encoded *Hyaluronan binding protein 4* (*Habp4)* (Figure 2B). Further bioinformatic analysis of *Serbp1* and *Habp4* suggested that *Serbp1* is expressed in mouse oocytes (Figure 2B), and that both genes belong to the hyaluronan/mRNA-binding protein family [22].

Next, we validated the physical interactions of SPIN1 with SERBP1 and HABP4 by performing co-immunoprecipitation assay in a heterologous expression system. When we pulled down MYC-tagged SPIN1 in cells co-expressing MYC-SPIN1 and HA-SERBP1, HA-tagged SERBP1 could be detected by Western blotting (Figure 2C). Moreover, there was no HA-tagged SERBP1 detected when the pull-down was done in control cells expressing either MYC-SPIN1 or HA-SERBP1, indicating the binding was specific. We further confirmed the interaction by detecting HA-tagged SPIN1 in MYC-tagged SERBP1 pull-downs prepared from cells co-expressing MYC-SERBP1 and HA-SPIN1 (Figure S2). Similar observations were made when immunoprecipitation assays were performed in cells co-expressing MYC-SPIN1 and HA-HABP4 (Figure 2D). In summary, we identified and validated the physical interactions of SPIN1 with hyaluronan/mRNA-binding protein family members: SERBP1 and HABP4.

### SPIN1 and SERBP1 are Co-expressed in Growing and Fully Grown Oocytes

**Figure 3:**
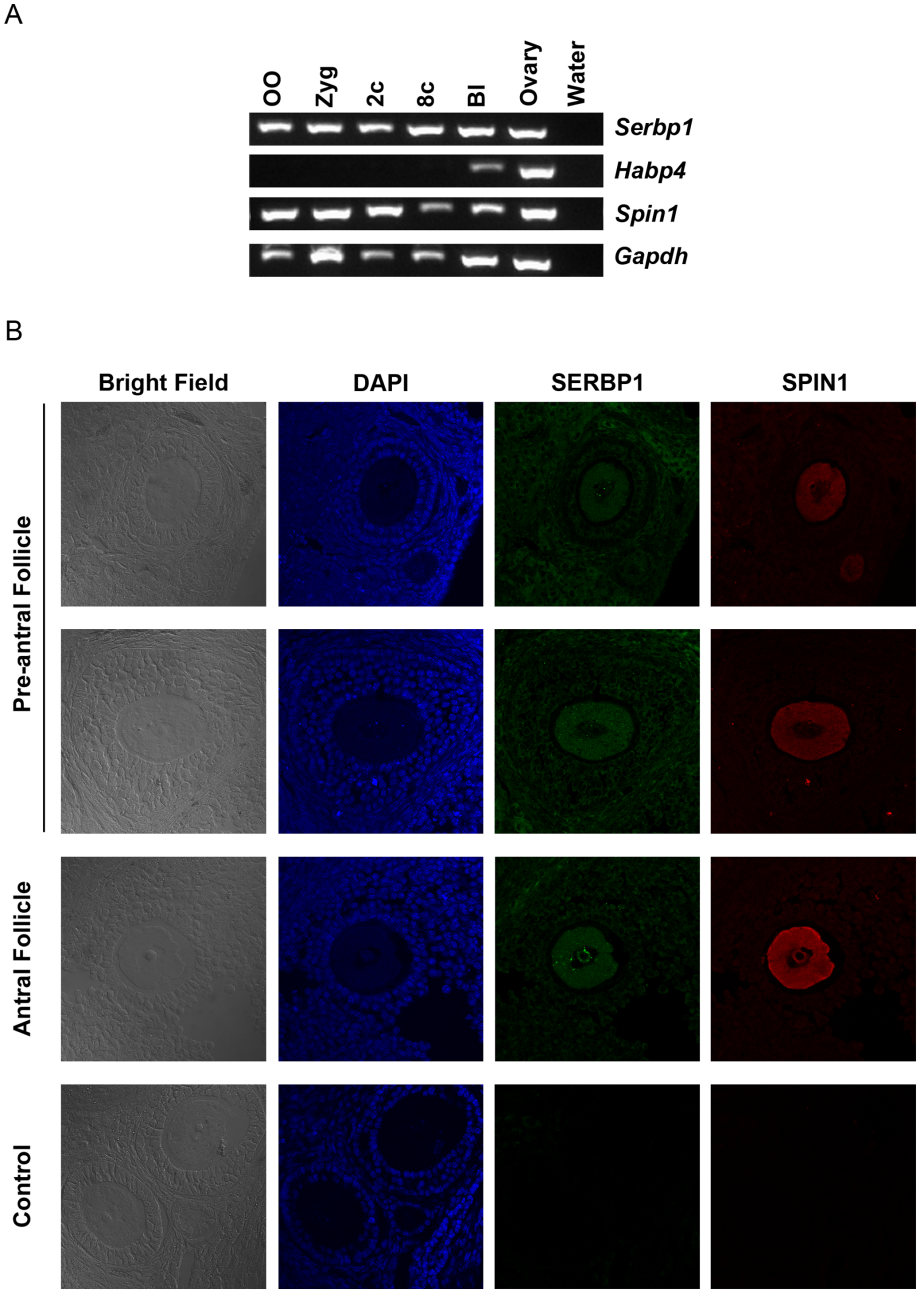
Expression profiles of *Spin1*, *Serbp1*, *and Habp4*. (A) Expression of *Spin1*, *Serbp1*, *Habp4*, and *Gapdh* in pre-implantation embryos and ovaries. OO: ovulated oocyte; Zyg: zygote; 2c: 2-cell stage embryo; 8c: 8-cell stage embryo; Bl: blastocyst. Each lane is the RT-PCR product of two embryos or two oocytes. Gapdh serves as controls. (B) Immunostaining of SPIN1 (red) and SERBP1 (green) on wild type ovarian sections by anti-SPIN1 and anti-SERBP1 as primary antibodies. Preimmune serum were used as controls. DNA (blue) was visualized by Hoechst 33342 dye.

Our findings that SPIN1 interacts physically with SERBP1 and HABP4 suggest these proteins are co-expressed in the same tissues or cells. To this end, we examined the expression patterns of *Spin1*, *Serbp1*, and *Habp4* in ovulated oocytes, pre-implantation embryos, and ovaries. *Serbp1* mRNA, similar to *Spin1* mRNAs, was detected in oocytes, zygotes, 2- and 8- cell embryos, blastocysts, and ovaries (Figure 3A). *Habp4* mRNA, however, was co-detected with *Spin1* mRNAs only in blastocyst-stage embryos and ovarian tissues (Figure 3A). Expression of *Spin1* and *Serbp1* in ovulated oocytes and early embryos suggests that these genes are maternally expressed, and deposited during oocyte growth. When expression of SPIN1 and SERBP1 was examined in ovarian follicles, by immunostaining ovarian tissue sections with antibodies recognizing SPIN1 and SERBP1, these two proteins were detected in follicles of pre-antral and antral stages (Figure 3B). In these follicles, SERBP1 was detected in granulosa cells in addition to oocytes, whereas SPIN1 was predominantly expressed in oocytes (Figure 3B). SPIN1 is detectable in the nucleolus of the oocyte in the antral follicle (Figure 3B) but not in the denuded fully grown oocyte (Figure 1C). This could be the result of the differences in sample treatment, since the oocytes in the antral follicle were examined after fixation and cryo-sectioning whereas the fully grown oocytes were fixed and permeabilized for whole-mount staining. Our expression analyses of *Spin1*, *Serbp1* and *Habp4* suggested that SPIN1 and SERBP1, but not HABP4, function as a protein complex in oocytes. Thus, in subsequent studies we focused on the SPIN1/SERBP1 interaction.

### SPIN1 Binds and Modulates RNA-binding Activity of SERBP1 via Tudor-like Domain

**Figure 4:**
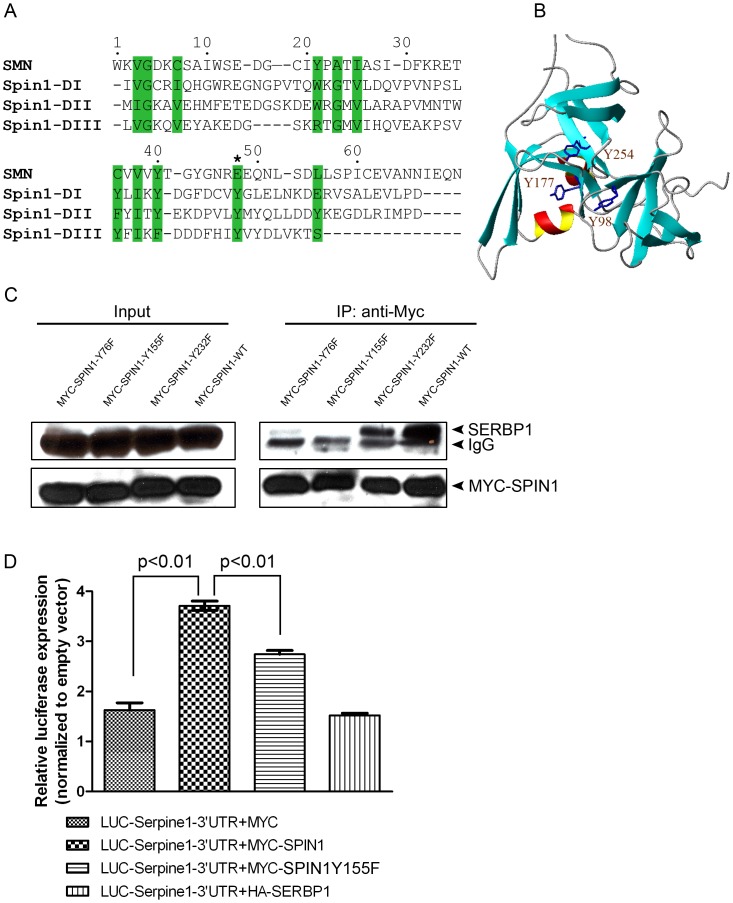
Tudor domains are required for SPIN1 functions. (A) Structural alignment of three SPIN1 Tudor domains with the SMN Tudor domain, adapted from [15]. Amino acid residues important for the structural fold of Tudor domain are highlighted in green. The glutamate residue involved in SMN protein-protein interactions is denoted by an asterisk. (B) Three-dimensional structure of human SPIN1 (PDB ID: 2NS2). The three tyrosine residues (Y98, Y177, Y254) of human SPIN1 are displayed. (C) Co-immunoprecipitation assay of SERBP1 with SPIN1 containing various mutations in the Tudor domains. HEK293T cells were transfected with MYC-tagged SPIN1 wild type or with the MYC-tagged SPIN1 point-mutants. The protein complexes were pulled down with MYC-antibody, and the endogenous SERBP1 was probed using SERBP1-antibody. (D) Luciferase reporter assay to measure the RNA-binding activity of SERBP1. HEK293T cells were transfected with luciferase reporters carrying the Serpine1 3′UTR, and were then co-transfected with the various plasmids. The RNA-binding activity of the endogenous SERBP1 was measured based on the relative expression of luciferase. Data are mean ± SEM, Student’s t-tests.

It has been reported that three Tudor-like domains, which recognize the methylated arginine or lysine residues in glycine/arginine-rich motif clusters (RGG/RXR box, where X is any amino acid) of the binding proteins, are contained in mouse and human SPIN1 [12], [15]. Consistently, SERBP1 has two conserved clusters of RGG/RXR motifs in which the arginine residues are methylated [22], [23]. To test whether the Tudor-like domains of SPIN1 are required to interact with SERBP1, we initially analyzed the structural alignments of the Tudor-like domains from human SPIN1, which have 99% identity to the Tudor-like domains of mouse SPIN1, and the Tudor domain of human Survival Motor Neuron protein (SMN1) [15]. It has been shown that the glutamate residue at position 134 within the SMN1 Tudor domain is crucial for protein-protein interactions [24]. This residue corresponds to tyrosine residues (Y98, Y177, and Y254) in the three human SPIN1 Tudor-like domains (Figure 4A). Interestingly, these three tyrosine residues are present on the surface of human SPIN1 protein (Figure 4B), thus available for protein-protein interactions. We then point-mutagenized these three corresponding tyrosine residues (Y76, Y155, and Y232) in the mouse SPIN1 Tudor domain to phenylalanines, and performed co-immunoprecipitation assay to determine the binding of these *Spin1* mutant proteins with SERBP1. Among the three *Spin1* mutant proteins, the binding of *Spin1*-Y155F mutant protein to SERBP1 was significantly affected, whereas *Spin1*-Y232F mutant protein retained the most binding capacity, and the tyrosine mutation within the first Tudor-like domain of SPIN1 partly abrogated its binding to SERBP1 (Figure 4C). Since all three *Spin1* mutant proteins were expressed at similar levels as the wild type SPIN1 (Figure 4C, Input), the failure of SPIN1 to interact with SERBP1 was not because of instability of the mutant proteins. Our study thus showed that formation of SPIN1/SERBP1 protein complexes is dependent on the Tudor-like domains of SPIN1.

**Figure 5:**
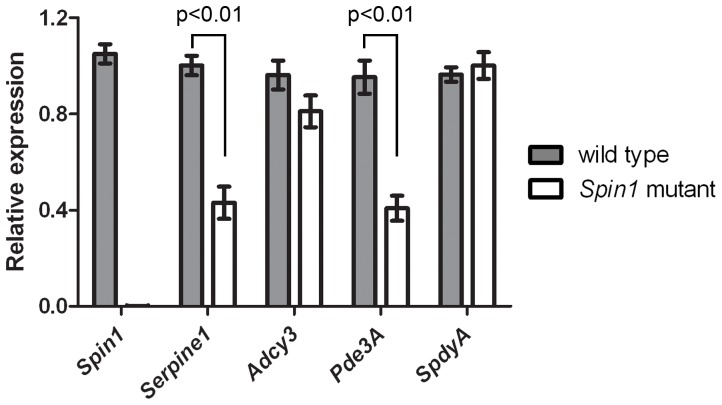
Maternal *Pde3A* mRNA is reduced in the *Spin1* mutant oocytes. Expression levels of *Spin1*, *Serpine1*, *Adcy3*, *Pde3A*, and *SpdyA* were measured by real-time quantitative PCR (qPCR) using mRNAs isolated from wild type- and *Spin1*-mutant fully grown oocytes, then reversed transcribed to cDNA. The cDNAs were pre-amplified prior to qPCR. Data are mean ± SEM, Student’s t-tests.

SERBP1 is an mRNA-binding protein that regulates mRNA stability of *Serpine1*, which is a plasminogen activator inhibitor (PAI-1). To determine the effects of SPIN1 and SERBP1 binding, we fused the 3′ untranslated region (UTR) of *Serpine1* mRNA, which is bound by SERBP1 [25], downstream of a firefly luciferase gene under a constitutive promoter. The firefly luciferase containing the *Serpine1* 3′UTR is expressed constitutively, but its mRNA stability and/or translation are controlled by SERBP1. This eventually determines the luciferase protein levels and activity. If SPIN1 binds and regulates SERBP1 mRNA-binding activity, over-production of SPIN1 may promote or dampen the expression of firefly luciferase. When we transfected firefly luciferase containing *Serpine1* 3′UTR into cells, luciferase activity was up-regulated by the presence of endogenous SERBP1 (Figure 4D). Interestingly, the luciferase activity was further enhanced upon over-production of wild type SPIN1. However, when a *Spin1* mutant protein defective in binding to SERBP1 (SPIN1-Y155F) was over-produced in these cells, the level of luciferase decreased (Figure 5D and S3). Luciferase activity was unchanged when SERBP1 instead of SPIN1 was over-produced, suggesting the enhancement was dependent on SPIN1 expression (Figure 4D and S3). Our results suggested that SPIN1 binds and regulates SERBP1 mRNA-binding activity, and the Tudor-like domain is required for this action.

### Maternal Transcript Stability is Affected in *Spin1* Mutant Oocytes

We showed that SPIN1 interacts with SERBP1 to regulate gene expression post-transcriptionally, and SPIN1 has a role in meiotic resumption in oocytes. Based on these findings, we hypothesized that SPIN1 regulates the mRNA stability of genes involved in resuming meiosis in the oocytes. To test this possibility, we examined the expression levels of maternal transcripts implicated in meiotic resumption: adenylyl cyclase 3 (*Adcy3*), phosphodiesterase 3A (*Pde3A*), and *Speedy A* (*SpdyA*) by using real-time quantitative PCR. Interestingly, the expression level of *Pde3A* mRNAs was reduced about 50% in *Spin1* mutant oocytes compared to controls, whereas *Adcy3* mRNA levels remained largely unchanged (Figure 5). The expression of a meiotic resumption inducer, *Speedy A* (*SpdyA*), was also not affected in *Spin1* mutant oocytes (Figure 5). Consistently, the SERBP1-regulated *Serpine1* mRNA showed reduced expression in *Spin1* mutant oocytes (Figure 5). Our studies suggested SPIN1 regulates maternal transcripts including one that encodes the cAMP-degrading enzyme PDE3A in oocytes.

## Discussion

*Spin1* is highly expressed in mammalian oocytes, and has been suggested to play a role in meiosis [14], [26]. Through characterizing a mouse mutant defective in *Spin1* and identifying its protein interacting partners, we have established that SPIN1 forms an RNP complex with the mRNA-binding protein SERBP1 and is involved in the resumption of meiosis in mammalian oocytes.

SPIN1 contains three Tudor-like domains [15], which are required for protein-protein interactions and its function (this study). Tudor domain proteins were first identified in flies as highly expressed genes in oocytes and play important roles in regulating female gametogenesis, mainly by participating in RNA processing and post-transcriptional regulatory processes [27]. Several Tudor domain proteins have been detected in the mouse oocyte, however their functions in the mammalian female germ cell have not been reported [28]. Our study suggests that the SPIN1 Tudor-like domain protein participates in female gametogenesis possibly by contributing to RNP complex formation in the mammalian oocyte. SPIN1 interacts with both SERBP1 and HABP4, which have been implicated in the regulation of RNA processes and translational control [22], [25], [29]. SERBP1 and HABP4 both contain the Tudor domain protein recognition motif, RGG/RXR [22]. However, SERBP1 and HABP4 are different in their expression pattern (this study) and protein sequences. Analyses of SERBP1 and HABP4 protein sequences show that both proteins are conserved at the C-terminus but not in their N-terminal and central regions [22]. This may allow different protein complex formation by these two proteins with SPIN1. Other than SERBP1 and HABP4 identified in this study, SPIN1 is also found in protein complexes such as those containing Histone H3 [16], [30] and Argonaute 3 [31] in mammalian cells, suggesting that SPIN1 functions as a recruitment domain in diverse cellular processes. Aberrant interaction with these gene products may lie at the root of the early post-natal lethality of *Spin1* mutants. Whether SPIN1 interactions with these proteins are also important in the oocyte remain to be tested.

Meiotic resumption relies largely on post-transcriptional regulation of maternal mRNAs stored in the fully grown oocyte. Messenger RNAs of several cell cycle regulators such as *Cyclin B1*, *Cdc25*, and *c-Mos* are kept dormant during oocyte growth and are translated in a timely fashion to initiate meiotic resumption [5], [10], [32], [33]. The finding that SPIN1/SERBP1 RNP regulates *Pde3A* mRNAs suggests that *Pde3A* may also be subject to translational control in oocytes. During the long period of meiotic arrest, PDE3A enzymatic activity in the oocyte is inhibited by transfer of cyclic guanine monophosphate (cGMP) from the surrounding granulosa cells, leading to accumulation of cAMP and prevention of meiotic resumption [34]. Upon a surge of luteinizing hormone (LH), or when oocytes are denuded of the granulosa cells, the inhibition of PDE3A activity is relieved in the oocyte as the levels of cGMP drop [35]. Active PDE3A then degrades cAMP to promote resumption of meiosis. The meiotic arrest phenotype of *Spin1* mutant oocytes may be attributed to the decreased level of *Pde3A* mRNA. It is possible that maternal *Pde3A* mRNA is continuously translated in the oocyte, ensuring a sufficient level of PDE3A during meiotic resumption, and a rapid response to the hormone signaling. Post-transcriptional control of *Pde3A* expression by the SPIN1/SERBP1 RNP complex in oocytes would ensure timely and efficient resumption of meiosis after long-term arrest.

SPIN1 and SERBP1 have been found in the protein complex composed of β-arrestins in mammalian cells [36]. β-arrestins are cytosolic proteins that participate in desensitization of G-protein-coupled receptors to dampen cellular responses to stimuli [37]. Mammalian oocytes express β-arrestin 2 and also a constitutively active G-protein-coupled receptor GPR3, which maintains high cAMP levels and meiotic arrest [38], [39]. This leads us to speculate that β-arrestin may couple post-transcriptional control through the SPIN1/SERBP1 RNP complex to desensitize GPR3 signaling in the oocyte, allowing meiotic resumption. Thus, SPIN1 may act as a scaffold protein via its Tudor-like domain for the transcriptionally inactive oocyte to modulate pathways, leading to meiotic resumption.

## Supporting Information

Figure S1 Characterization of Spin1 genetrap mouse line. (A) Genomic organization of mouse *Spindlin1 (Spin1)*. *Spin1* exons 1–6 are depicted by white boxes. β-geo denotes the cassette containing the galactosidase/neomycin phosphotransferase fusion gene. (B) Genotyping results of wild type (+/+), *Spin1* genetrap heterozygous (GT/+) and homozygous E18.5 fetuses (GT/GT). (C) RT-PCR analysis of *Spin1* expression in wild type- and *Spin1*-mutant E18.5 fetal gonads. Gapdh was included as loading controls. (D) Protein expression analysis of *Spin1* in wild type and *Spin1* mutant E18.5 fetal gonads by Western blotting.
(DOCX)

Figure S2 MYC-tagged SERBP1 is co-immunoprecipitated with HA-tagged SPIN1. MYC-tagged SERBP1 was pulled down from HEK293T cells using MYC-antibody. HA-tagged SPIN1 was probed using HA-antibody.
(DOCX)

Figure S3 Wild type SPIN1 and SPIN1 point mutant (Y155F) are expressed at similar level in HEK293T cells. Protein extracts were prepared from HEK293T cells expressing various constructs. Endogenous SERBP1 and MYC-SPIN1 were probed with SERBP1-antibody and MYC-antibody, respectively. Actin was included as loading control.
(DOCX)

Table S1 (DOCX)

Table S2 (DOCX)

Table S3 (DOCX)

Table S4 (DOCX)
