# Supplementary figures and images for: A Tudor Domain Protein SPINDLIN1 Interacts with the mRNA-Binding Protein SERBP1 and Is Involved in Mouse Oocyte Meiotic Resumption
Source: PLoS One. 2013 Jul 22;8(7):e69764. doi: 10.1371/journal.pone.0069764 (PMC3718791; doi:10.1371/journal.pone.0069764)

**Figure S1**

**
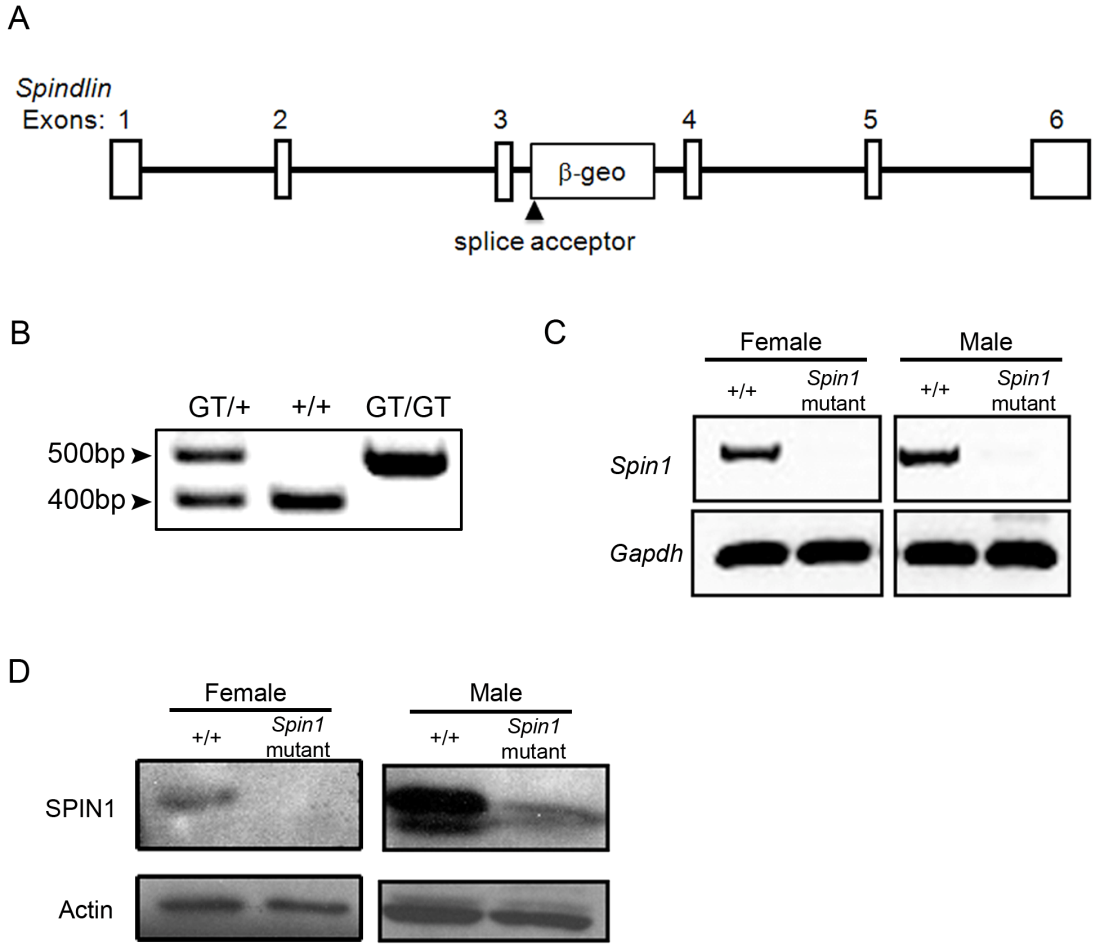
**

Supplement: Figure S1 — Characterization of Spin1 genetrap mouse line. (A) Genomic organization of mouse Spindlin1 (Spin1). Spin1 exons 1–6 are depicted by white boxes. β-geo denotes the cassette containing the galactosidase/neomycin phosphotransferase fusion gene. (B) Genotyping results of wild type (+/+), Spin1 genetrap heterozygous (GT/+) and homozygous E18.5 fetuses (GT/GT). (C) RT-PCR analysis of Spin1 expression in wild type- and Spin1-mutant E18.5 fetal gonads. Gapdh was included as loading controls. (D) Protein expression analysis of Spin1 in wild type and Spin1 mutant E18.5 fetal gonads by Western blotting. (DOCX) [file pone.0069764.s001.docx]

**Figure S2**

**
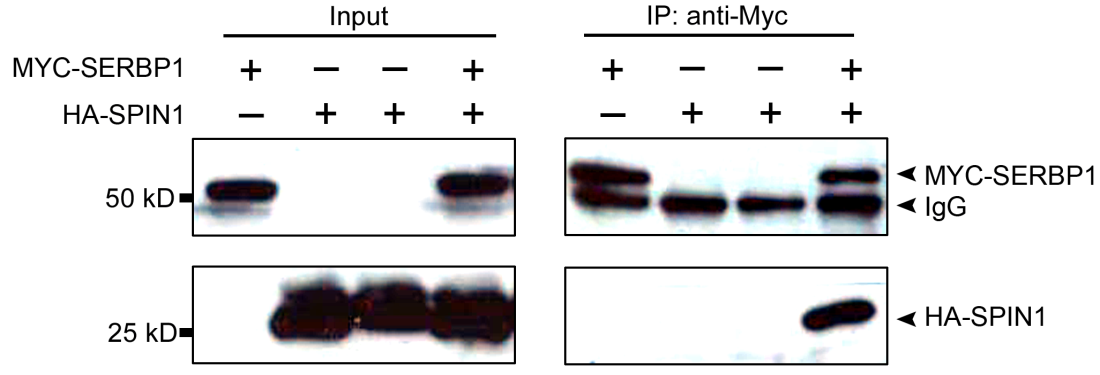
**

Supplement: Figure S2 — MYC-tagged SERBP1 is co-immunoprecipitated with HA-tagged SPIN1. MYC-tagged SERBP1 was pulled down from HEK293T cells using MYC-antibody. HA-tagged SPIN1 was probed using HA-antibody. (DOCX) [file pone.0069764.s002.docx]

**Figure S3**

**
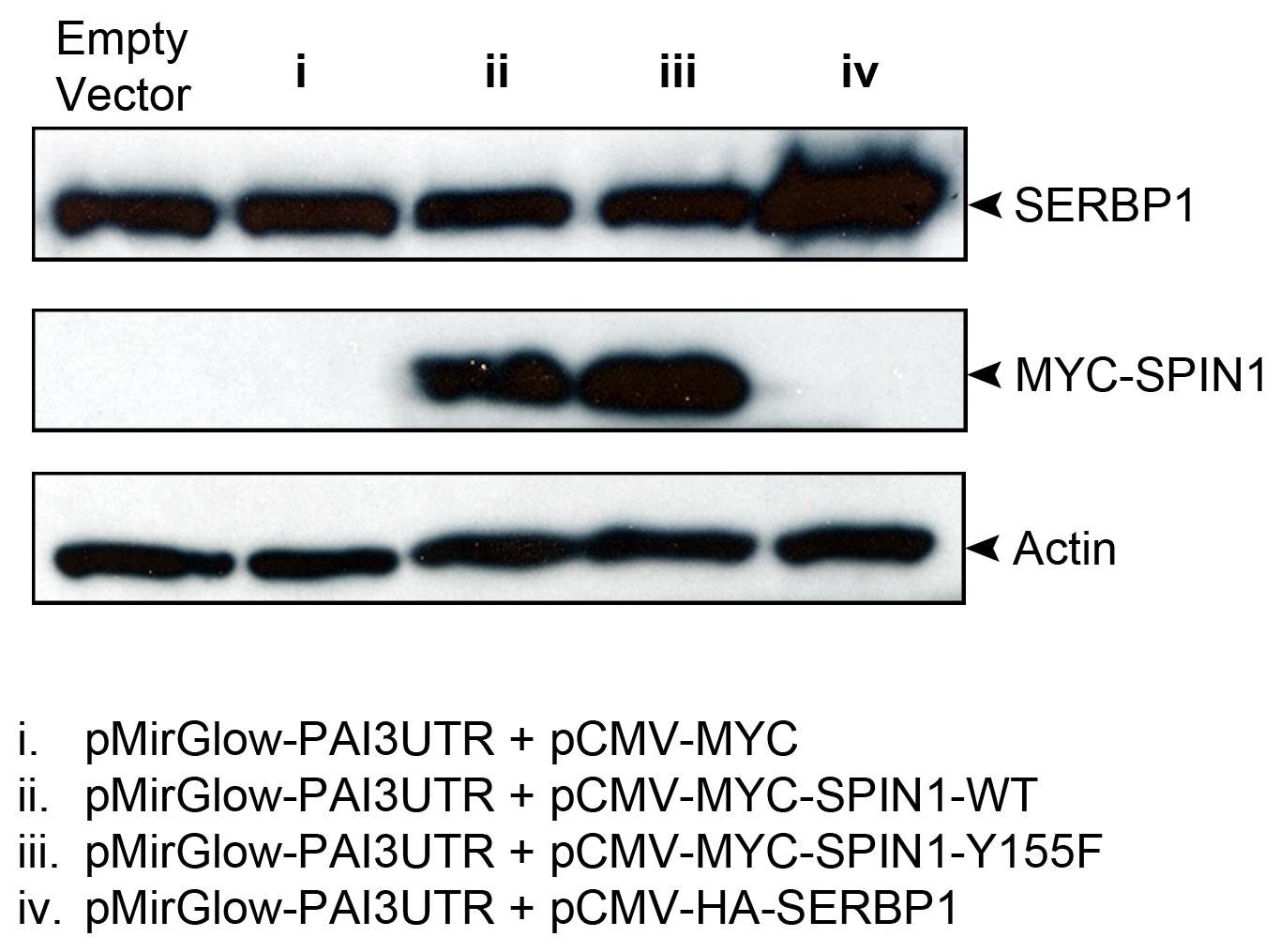
**

Supplement: Figure S3 — Wild type SPIN1 and SPIN1 point mutant (Y155F) are expressed at similar level in HEK293T cells. Protein extracts were prepared from HEK293T cells expressing various constructs. Endogenous SERBP1 and MYC-SPIN1 were probed with SERBP1-antibody and MYC-antibody, respectively. Actin was included as loading control. (DOCX) [file pone.0069764.s003.docx]
