# Supplementary material for: A Tudor Domain Protein SPINDLIN1 Interacts with the mRNA-Binding Protein SERBP1 and Is Involved in Mouse Oocyte Meiotic Resumption
Source: PLoS One. 2013 Jul 22;8(7):e69764. doi: 10.1371/journal.pone.0069764 (PMC3718791; doi:10.1371/journal.pone.0069764)
Supplement: Table S1 — (DOCX) [file pone.0069764.s004.docx]

**Table S1. Primers used in pCMV6-AN cloning**

| **Primer number** | **Sequences (5’ to 3’)** | **Remarks** |
| --- | --- | --- |
| COH037 | 5’ TTG GCG CGC CAG CCT CTG CGT CAA GTC CTG CTT C 3’ | Forward primer to amplify *Spin1* |
| COH004 | 5’ ATA AGA ATG CGG CCG CCT AGG ATG TTT TCA CCA AAT CGT A 3’ | Reverse primer to amplify *Spin1* |
| COH070 | 5’ TTG GCG CGC CAC CTG GGC ACC TAC AGG AAG GCT TC 3’ | Forward primer to amplify *Serbp1* |
| COH071 | 5’ ATA AGA ATG CGG CCG CTT AGG CCA GAG CTG GGA AGG CCT C 3’ | Reverse primer to amplify *Serbp1* |
| COH240 | 5’ TTG GCG CGC CAC GAA GGT CCT ACA GGG AAT AC 3’ | Forward primer to amplify *Habp4* |
| COH237 | 5’ ATA AGA ATG CGG CCG CTT AGG CCA GGG CGG GGA AGT CC 3’ | Reverse primer to amplify *Habp4* |
