# Supplementary material for: A Tudor Domain Protein SPINDLIN1 Interacts with the mRNA-Binding Protein SERBP1 and Is Involved in Mouse Oocyte Meiotic Resumption
Source: PLoS One. 2013 Jul 22;8(7):e69764. doi: 10.1371/journal.pone.0069764 (PMC3718791; doi:10.1371/journal.pone.0069764)
Supplement: Table S2 — (DOCX) [file pone.0069764.s005.docx]

**Table S2. Primers used in site-directed mutagenesis of *Spin1***

| **Primer number** | | **Sequences (5’ to 3’, mutated nucleotides are highlighted in red)** | **Remarks** |
| --- | --- | --- | --- |
| COH117 | 5’ GTA CGA TGG ATT TGA CTG TGT TTT TGG ACT AGA ACT TAA TAA GGA TG 3’ | | Forward primer to point mutagenize the 1^st^ tyrosine in the SPIN1 Tudor-like domain to phenylalanine |
| COH118 | 5’ CAT CCT TAT TAA GTT CTA GTC CAA AAA CAC AGT CAA ATC CAT CGT AC 3’ | | Reverse primer to point mutagenize the 1^st^ tyrosine in the SPIN1 Tudor-like domain to phenylalanine |
| COH119 | 5’ ATG AGA AAG ACC CTG TCT TGT TCA TGT ACC AGC TCC TCG ATG AC 3’ | | Forward primer to point mutagenize the 2^nd^ tyrosine in the SPIN1 Tudor-like domain to phenylalanine |
| COH120 | 5’ GTC ATC GAG GAG CTG GTA CAT GAA CAA GAC AGG GTC TTT CTC AT 3’ | | Reverse primer to point mutagenize the 2^nd^ tyrosine in the SPIN1 Tudor-like domain to phenylalanine |
| COH121 | 5’ GAT GAC GAT TTC CAT ATT TTC GTC TAC GAT TTG GTG AAA ACA TC 3’ | | Forward primer to point mutagenize the 3^rd^ tyrosine in SPIN1 Tudor-like domain to phenylalanine |
| COH122 | 5’GAT GTT TTC ACC AAA TCG TAG ACG AAA ATA TGG AAA TCG TCA TC 3’ | | Reverse primer to point mutagenize the 3^rd^ tyrosine in SPIN1 Tudor-like domain to phenylalanine |
