# Supplementary material for: A Tudor Domain Protein SPINDLIN1 Interacts with the mRNA-Binding Protein SERBP1 and Is Involved in Mouse Oocyte Meiotic Resumption
Source: PLoS One. 2013 Jul 22;8(7):e69764. doi: 10.1371/journal.pone.0069764 (PMC3718791; doi:10.1371/journal.pone.0069764)
Supplement: Table S3 — (DOCX) [file pone.0069764.s006.docx]

**Table S3. Primers used in pmirGLO cloning**

| **Primer number** | | **Sequences (5’ to 3’)** | **Remarks** |
| --- | --- | --- | --- |
| COH265 | 5’ AGC TTT GTT TAA ACC CAA ATG TGG GGT CAC ATG GTC 3’ | | Forward primer to amplify rat *Serpine1* 3’UTR |
| COH266 | 5’ TGC TCT AGA GTC TGC ATA TTC TCT ATT TTT ATT A 3’ | | Reverse primer to amplify rat *Serpine1* 3’UTR |
