# Supplementary material for: A Tudor Domain Protein SPINDLIN1 Interacts with the mRNA-Binding Protein SERBP1 and Is Involved in Mouse Oocyte Meiotic Resumption
Source: PLoS One. 2013 Jul 22;8(7):e69764. doi: 10.1371/journal.pone.0069764 (PMC3718791; doi:10.1371/journal.pone.0069764)
Supplement: Table S4 — (DOCX) [file pone.0069764.s007.docx]

**Table S4. Primers used in real time qPCR analysis**

| **Primer number** | | **Sequences (5’ to 3’)** | **Remarks** |
| --- | --- | --- | --- |
| COH313 | 5’ TCA CCT ATG AGA AAG ACC CTG TC 3’ | | Forward primer to probe *Spin1* |
| COH314 | 5’ CTG CTG GAG GCG AAT CAT 3’ | | Reverse primer to probe *Spin1* |
| COH293 | 5’ AGG ATC GAG GTA AAC GAG AGC 3’ | | Forward primer to probe *Serpine1* |
| COH294 | 5’ GCG GGC TGA GAT GAC AAA 3’ | | Reverse primer to probe *Serpine1* |
| COH457 | 5’ GCT CAA TGG CAC TGA CAG G 3’ | | Forward primer to probe *Adcy3* |
| COH458 | 5’ ATG ACG AAC ATC ATC ACA GTC A 3’ | | Reverse primer to probe *Adcy3* |
| COH441 | 5’ CCC AGA CCA TGA TCT TCC TG 3’ | | Forward primer to probe *Pde3A* |
| COH442 | 5’ CCA GGT GTT CAA CTG ATC CA 3’ | | Reverse primer to probe *Pde3A* |
| COH453 | 5’ GCC TGA GGT AGA TCG CTG AG 3’ | | Forward primer to probe *SpdyA* |
| COH454 | 5’ CGC ATT TTA GCT TCC CAA GA 3’ | | Reverse primer to probe *SpdyA* |
| COH000 | 5’ AGC TTG TCA TCA ACG GGA AG 3’ | | Forward primer to probe *Gapdh* |
| COH000 | 5’ TTT GAT GTT AGT GGG GTC TCG 3’ | | Reverse primer to probe *Gapdh* |
| COH481 | 5’ TGG ACC ATA GAA CTC ACA GTA CCT 3’ | | Forward primer to probe 3’UTR of *Serpine1* |
| COH482 | 5’ TGG GAA CTG GGT CCA AAG 3’ | | Reverse primer to probe 3’UTR of *Serpine1* |
| COH473 | 5’ ACT CGT ATT CAC GCA TGC AC 3’ | | Forward primer to probe 3’UTR of *Pde3A* |
| COH474 | 5’ ACC TTG TGG AGT CAG GCA TC 3’ | | Reverse primer to probe 3’UTR of *Pde3A* |
